# Visual Search in Chinese Children With Attention Deficit/Hyperactivity Disorder and Comorbid Developmental Dyslexia: Evidence for Pathogenesis From Eye Movements

**DOI:** 10.3389/fpsyg.2020.00880

**Published:** 2020-05-20

**Authors:** Xiaohui Cui, Jiuju Wang, Yulin Chang, Mengmeng Su, Hannah T. Sherman, Zhaomin Wu, Yufeng Wang, Wei Zhou

**Affiliations:** ^1^Beijing Key Laboratory of Learning and Cognition, School of Psychology, Capital Normal University, Beijing, China; ^2^Institute of Mental Health, Peking University Sixth Hospital, Beijing, China; ^3^National Clinical Research Center for Mental Disorders and Key Laboratory of Mental Health, Ministry of Health, Peking University, Beijing, China; ^4^College of Elementary Education, Capital Normal University, Beijing, China; ^5^Massachusetts General Hospital, Harvard Medical School, Charlestown, SC, United States; ^6^Shenzhen Children’s Hospital, Shenzhen, China

**Keywords:** attention deficit/hyperactivity disorder with comorbid developmental dyslexia, eye tracking, visual search, Chinese character, pathogenesis

## Abstract

In this study, a visual search task was conducted on children with comorbid attention deficit/hyperactivity disorder (ADHD) and developmental dyslexia (DD), children with pure ADHD, and typically developing children to explore the pathogenesis of comorbidity between ADHD and DD. Participants searched for the target character from five characters in each trial during the task. The distractors included orthographically similar characters, homophones, unrelated characters, and characters of a different color (i.e., red). Results showed that the clinical groups produced longer first fixation duration than the control group in all types of distractors. Children with ADHD comorbid DD were also more susceptible to characters with the distracting red color in gaze duration and total viewing time than were children with pure ADHD and healthy controls. The implication of comorbidity (ADHD + DD) on the pathogenesis was discussed. These results may be helpful for the diagnosis and treatment of ADHD with comorbid DD.

## Introduction

Although much literature documents significant comorbidity (about 20–40%) between attention deficit/hyperactivity disorder (ADHD) and developmental dyslexia (DD) (Friedman et al., 2010; Germanò et al., 2010), the etiology of this comorbidity remains unclear (Caron and Rutter, 1991; Neale and Kendler, 1995; Willcutt et al., 2010). The shared cognitive deficit model suggests that one possible reason for the co-occurrence of two disorders is that they share the same cognitive overactivity (Caron and Rutter, 1991), whereas the distinguished cognitive deficit model considers the comorbidity as an independent disorder. The implication seems to be that the comorbidity pattern is constituted by either a variety of disorders as a superposition or a meaningfully distinctive syndrome on its own (Neale and Kendler, 1995).

Although the core deficit of ADHD and DD was believed to be attention and language, respectively (Weiler et al., 2002), some studies have reported that children with ADHD also showed deficits in letter or word recognition (Wei et al., 2014; Lúcio et al., 2017; Mohammadhasani et al., 2019) and that DD children also demonstrated deficits in visual attention or response inhibition (Willcutt et al., 2000; Martin et al., 2006; Vidyasagar and Kristen, 2010). These results may provide evidence of the shared cognitive deficit model. Willcutt et al. (2001) used a sample (*n* = 102) of twins to investigate the phoneme awareness and executive functioning performance of individuals of ADHD and DD. Results indicated that individuals with ADHD comorbid DD did not exhibit more severe reading difficulties than those with DD alone. They also did not exhibit more symptoms of ADHD than those with ADHD alone. The following research (Willcutt et al., 2005) also showed that groups with ADHD and with DD both exhibited weakness in reading skills, verbal working memory, processing speed, and response inhibition. Recently, the automatic deficit hypothesis proposes that children with ADHD also show impairments in automatic (fast, effortless, and autonomous) processes (Fabio, 2017; Martino et al., 2017; Caprì et al., 2020), in addition to controlled (effortful, slow, and prone to errors) processes. These impairments in automatic processes could be identified in children with DD as well (Lum et al., 2012).

On the other hand, some studies have found unique deficits in participants with ADHD comorbid DD. Rucklidge and Tannock (2002) found that individuals with ADHD comorbid DD had worse performance in color naming in the Stroop task than had individuals with pure ADHD. Purvis and Tannock (2000) used a series of inhibition and phonological tasks and found that participants with ADHD comorbid DD generally exhibited the deficits of both the pure ADHD and pure DD groups in an additive fashion, which matched the distinguished cognitive deficit model. It remains unclear which model explains the pathogenesis of comorbidity disorder.

Various models in the pathogenesis of ADHD comorbid DD have been supported by previous studies that used different clinical participants and tasks. Although many previous studies have investigated the deficits of ADHD or DD separately, few studies have included the comorbid (ADHD + DD) group. Neither the shared cognitive deficit model nor the distinguished cognitive deficit model can be examined without considering the comorbid group. The present study would examine the difference between the pure ADHD and comorbid (ADHD + DD) groups. Investigation on the comorbidity between ADHD and DD mainly included two core cognitive domains, attention/inhibition and language processing, which were usually not examined at the same time. For instance, using only linguistic tasks (orthographic decision and phonological decision task, and rapid automatized naming), De Jong et al. (2012) found that DD, ADHD, and the comorbidity between the two disorders presented similar impairments, which supported the shared cognitive deficit model. However, the distinguished cognitive deficit model can only be thoroughly tested when both attention/inhibition and language processing are examined. The present study aimed to test the etiological hypotheses of comorbidity through the task of visual search for Chinese characters (see Zhou et al., 2017 for the similar method). Typical visual search tasks use non-verbal items as the target, which assesses the mechanisms mediating selective attention and inhibition in vision (Mason et al., 2003). Our novelty paradigm manipulated the language (whether the distracting Chinese characters for visual search are orthographically or phonologically related to the target character) and attention/inhibition (whether the distracting Chinese characters have extraneous color) factors in one experiment, so that we would be able to compare both models in the same framework.

Compared with traditional methods, eye-movement technology can acquire more physiological indicators and information, which helps us analyze the reading and visual search processes objectively in real time. For example, Gould et al. (2001) used the eye movement techniques and found out that children with ADHD made larger saccades that interrupted fixation than did the control children, which might be helpful in making a clinical diagnosis of ADHD. Türkan et al. (2016) evaluated visual search patterns and the change detection performance in children with ADHD in the eye-movement study. They found out that compared with children with ADHD, the healthy controls made longer fixations on the changing area. These findings confirm that children with ADHD present difficulty in sustaining attention, which is necessary for encoding the scene properties and goal-oriented behavior (Türkan et al., 2016). Mohammadhasani et al. (2019) compared the eye-movement patterns during word list reading and memory tests in ADHD and typically developing children. They found that the visual scanning of ADHD individuals was discontinuous, uncoordinated, and chaotic in comparison with that in typically developing children. Researchers found that children with DD tended to have longer fixation times, shorter saccade amplitude, and more frequent return sweep than did the control group in both reading and visual search experiments (Garzia et al., 1990; Rayner, 1998). Deans et al. (2010) found that variables from eye tracking, such as the number of fixations, fixation duration, and total reading time, may have potential in differentiating clinical and control groups.

It has been found that participants with ADHD or DD demonstrate deficits in visual search for figures (Iles et al., 2000; Mullane and Klein, 2008), and this process is typically completed by attention processes and a series of eye movements (Treisman and Gelade, 1980). The present study would further compare the eye-movement patterns in the visual search task among children with ADHD comorbid DD, children with pure ADHD, and typically developing children. In addition, we used characters as visual search items so that linguistic and attentional processing could be examined simultaneously. Specifically, the visual search task required participants to search for the target characters with orthographically similar, homophonic, red color, and unrelated characters as distractors. Among these, similar Chinese characters and homophones correspond to linguistic processing, red color characters to attentive/inhibitive processing, and irrelevant characters to general processing.

For pathogenesis of comorbidity between ADHD and DD, the results would support the shared cognitive deficit model if both the comorbidity and pure ADHD groups had similar patterns in language and attention/inhibition processes and were significantly different than the control group. If the performance of the comorbid group was different from the pure ADHD group for either of the two processes, the results would be in line with the distinguished cognitive deficit model. When we examined various eye-movement measures, the prediction could be hierarchical, as first fixation duration (the duration of the first fixation that was within the current interest area), gaze duration (the summation of the duration across all fixations during the first run within the current interest area), and total viewing time (the total dwell time on the current interest area) reflect different processing stages. While the first fixation duration reflects early processing and attention attraction on the stimuli, gaze duration and total viewing time indicate a relatively late stage for information integration and attention maintenance.

## Materials and Methods

### Participants

A total of 45 children (all boys, *M*_age_ = 9.3, SD = 1.1) were recruited to participate in the study. The three groups were aged matched (*p* > 0.05). The ADHD group, the ADHD + DD comorbidity group, and the control group each contained 15 individuals. The diagnoses of ADHD and/or other psychiatric disorders were made through clinical and semi-structured interviews by child psychiatrists in the child psychiatric clinics at Peking University Sixth Hospital/Institute of Mental Health. The clinical diagnoses were made based on the *Diagnostic and Statistical Manual of Mental Disorders, 4th Edition* (DSM-IV) diagnostic criteria of ADHD (American Psychiatric Association,, 2000); and the semi-structured interviews were performed using the Schedule for Affective Disorders and Schizophrenia for School-Age Children-Present and Lifetime version (K-SADS-PL). Because there is currently no valid diagnostic tool for DD in the Chinese language, the diagnosis of DD was made according to the prior work of Shu et al. (2006) and Liu et al. (2017). Briefly, the participants were diagnosed as DD when their performance in Chinese character recognition or word list reading test was 1.5 standard deviations below the norm. The control group included typically developing pupils from the primary schools in Beijing and Shandong in China. None of the children in the control group had been diagnosed with any type of current or past major psychiatric disorders in the K-SADS-PL assessment; neurological disorders; or vision-related, reading-related, or attention-related deficiencies.

All groups of children (1) were right-handed, and their hearing and vision (or corrected vision) were normal; (2) had had an IQ above 80 in the Wechsler Intelligence test; (3) had no history of head injury with loss of consciousness or had no brain trauma or any kind of neurological disease; (4) had no prior history or current diagnosis of schizophrenia, affective disorder, Tourette syndrome, pervasive developmental disorder, or intellectual disability; and (5) had no history of drug or substance abuse. Informed consent was obtained from parents of children before the study. This study was conducted in accordance with the Declaration of Helsinki as revised in 1989 and approved by the Ethics Committee of Peking University Sixth Hospital.

### Materials

In each trial, five Chinese characters, including one target and four interferential characters, appeared on the screen for visual search. The interferential characters, as compared with the target characters, were orthographically similar, were homophonic, were unrelated, or of a different color (a different red character). The strokes (*M* = 4.9) and frequencies (*M* = 3,129 per million; Beijing Language Institute Publisher, 1986) of the target character and four types of interferential characters were matched (*p* > 0.05).

### Procedure

**FIGURE 1:**
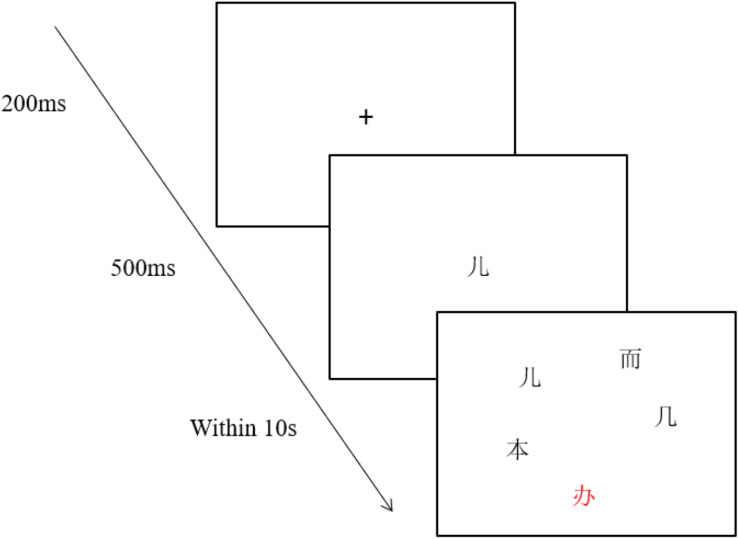
An example of experimental procedure. The character “
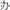
” is in red color. The other characters are in black color.

The experimental instrument was the EyeLink 1000 eye tracker made by SR Research in Canada. The stimulus appeared on a 21-inch Dell display screen with a resolution of 1,024 × 768, and the data acquisition frequency was 1,000 Hz. The participants sat 60 cm away from the screen, and their right eye movements were recorded. Before the experiment, the participants’ heads were fixed so that the experimenter could calibrate the participants’ eyes to establish the connection between their eyes and the computer. The practice test would start after five-point calibration and validation. During the experiment, a fixation “+” would appear in the center of the screen (see Figure 1). A participant’s fixation on the “+” would trigger the presentation of a target character. The central screen presented the target word for 500 ms. Then five characters (the target, orthographically similar, homophonic, unrelated, and red) would appear on the screen for 10 s. Each item occupied a 60 × 60 pixel grid. Participants had to concentrate on the screen and quickly select the target with the mouse. Each participant did a total of 60 trials after two exercises and was prompted to rest every 20 trials.

### Data Analysis

Fixations were determined with the default algorithm and parameters for saccade detection in EyeLink ELCL (the saccade motion threshold was 0.15° of visual angle, the saccade acceleration threshold was 8,000°/s^2^, and the saccade velocity threshold was 30°/s). We analyzed two global eye-movement measures [interregional saccade counts (the number of saccades among different characters) and saccade length] and four character-region-based eye-movement measures (first fixation duration, gaze duration, total viewing time, number of fixations). The behavioral indicators were collected as response latency and accuracy. We entered participants’ eye-movement measures in a 3 (group, between-participants) × 5 (type, within-participants) mixed model by repeat ANOVA and their interregional saccade number, saccade length, reaction time, and accuracy in one-way ANOVA. The age was added as a covariate in all repeated ANOVAs. If Mauchly’s Test of Sphericity indicated that the assumption of sphericity had been violated in the analysis, the results would be corrected (i.e., *p*_corrected_) using Greenhouse–Geisser estimates. Least significant difference (LSD) method was used during all pairwise comparisons.

## Results

Participants’ behavioral and eye-movement measures are shown in Tables 1, 2. In all conditions, there tended to be a worse performance in clinical children (individuals with ADHD both with and without DD) than in controls. The one-way ANOVA showed that the difference in the accuracies among the three groups of participants was significant, *F*(2, 42) = 6.683, *p* = 0.003, η^2^ = 0.241. *Post hoc* pairwise comparisons suggested that the accuracy of the comorbidity group (*p* = 0.006) and ADHD group (*p* = 0.025) was partially different than that of the control group. The group effect on reaction time showed a numerical trend, *F*(2, 42) = 2.913, *p* = 0.065, η^2^ = 0.122, which indicated that the comorbidity group produced longer reaction time than did the other two groups (*p*s < 0.05). Group effect on interregional saccade number was significant, *F*(2,42) = 5.213, *p* = 0.009, and *post hoc* comparisons showed that the comorbidity group had more interregional saccade numbers than had the ADHD and control groups. However, the group effect on mean saccade length is not statistically significant (*p* > 0.05).

**TABLE 1:** Descriptive statistical results of behavioral indicators of different groups and global eye-movement measures.

	Comorbidity	ADHD	Control	Results of the statistics
Accuracy	0.94 (0.03)	0.96 (0.02)	0.98 (0.01)	Control > ADHD = ADHD + DD
Reaction time	2706 (760)	2236 (529)	2260 (473)	ADHD + DD > ADHD = Control
Interregional saccade	4.7 (1.1)	3.8 (0.5)	3.9 (0.6)	ADHD + DD > ADHD = Control
Saccade length	184 (27)	190 (23)	175 (16)	ADHD + DD = ADHD = Control

*Standard deviations are shown in parentheses. ADHD, attention deficit/hyperactivity disorder; DD, developmental dyslexia.*

**TABLE 2:** Descriptive statistical results of character-region-based eye-movement indicators under various types of characters of different groups.

Indicators	Group	TAR	ORT	HOM	UNR	RED	Group main effect
FFD	Comorbidity	323 (71)	225 (39)	188 (34)	182 (33)	200 (49)	ADHD + DD =
	ADHD	326 (68)	232 (43)	179 (37)	181 (31)	175 (27)	ADHD > Control
	Control	274 (53)	207 (25)	164 (21)	164 (22)	158 (30)	
	Comorbidity	560 (167)	244 (34)	193 (37)	194 (59)	209 (54)	ADHD + DD > ADHD = Control
GD	ADHD	458 (144)	245 (45)	182 (41)	185 (32)	182 (37)	
	Control	373 (138)	214 (29)	165 (21)	165 (24)	161 (33)	
TT	Comorbidity	1032 (406)	367 (63)	243 (56)	243 (84)	296 (99)	ADHD + DD > ADHD = Control
	ADHD	819 (289)	374 (70)	234 (58)	223 (40)	224 (56)	
	Control	664 (285)	313 (51)	190 (35)	196 (35)	185 (40)	
NFIX	Comorbidity	2.7 (1.2)	1.1 (0.3)	0.5 (0.2)	0.5 (0.2)	0.3 (0.2)	ADHD + DD >
	ADHD	2.0 (0.7)	1.0 (0.3)	0.6 (0.1)	0.5 (0.1)	0.3 (0.2)	ADHD = Control
	Control	1.9 (0.8)	0.9 (0.2)	0.4 (0.1)	0.5 (0.1)	0.3 (0.1)	

*Standard deviations are shown in parentheses. FFD, first fixation duration; GD, gaze duration; TT, total view time; NFIX, number of fixations; TAR, target; ORT, orthographically similar; HOM, homophonic; UNR, unrelated.*

### First Fixation Duration

The results showed that there was a main effect of the group factor, *F*(2, 41) = 3.551, *p* = 0.038, η^2^ = 0.148. The first fixation duration of the comorbidity group (*p* = 0.014) and the ADHD group (*p* = 0.070) was longer than that of the control group, whereas the former two groups were not significantly different from each other (*p* > 0.05). There was also a significant main effect of the material type, *F*(4, 164) = 7.822, *p*_corrected_ < 0.001, η^2^ = 0.160. Participants’ first fixation duration of target characters was longer than that of distractors (*p*s < 0.001). The first fixation duration of orthographically similar characters was longer than homophonic, unrelated, and red characters (*p*s < 0.001), whereas the fixation duration of the latter three conditions was not significantly different from each other (*p*s > 0.05). There was no interaction between group and type (*p* > 0.05).

### Gaze Duration

There was a main effect of the group factor, *F*(2, 41) = 8.173, *p* = 0.001, η^2^ = 0.285. The comorbidity group had significantly longer gaze duration than the ADHD group (*p* = 0.027) and control group (*p* < 0.001). The difference between the ADHD and control group was not statistically significant (*p* > 0.05). There was a significant main effect of the material types, *F*(4, 164) = 12.391, *p*_corrected_ < 0.001, η^2^ = 0.232. Participants’ gaze duration of target characters was longer than that of distractors (*p*s < 0.001). The gaze duration of orthographically similar characters was longer than that of homophonic, unrelated, and red characters (*p*s < 0.001), but the latter three conditions had no significant differences (*p*s > 0.05). The interaction between group and type was significant, *F*(8, 164) = 4.561, *p*_corrected_ = 0.009, η^2^ = 0.182. The simple effect test showed that under all distracting conditions except for unrelated character, the gaze duration of the comorbidity group was significantly higher than that of the control group (*p*s < 0.05), whereas the ADHD group had no difference than either of the two groups (*p*s > 0.05) under all distracting conditions.

### Total Viewing Time

**FIGURE 2:**
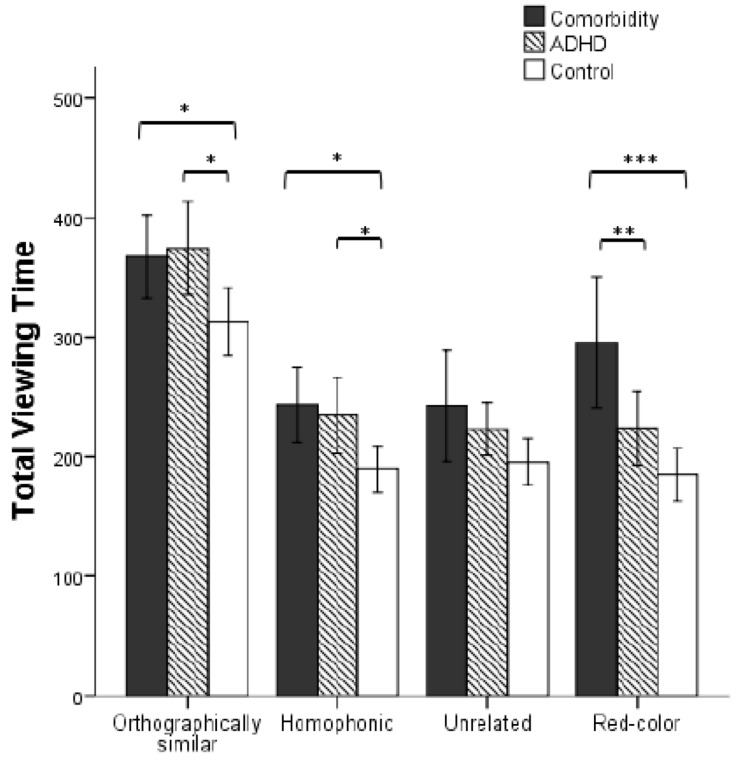
Three groups participants’ total viewing time under different types (error bars denote 95% CI. ^∗^*p* < 0.05, ^∗∗^*p* < 0.01, and ^∗∗∗^*p* < 0.005, respectively).

There was a main effect of the group factor, *F*(2, 41) = 9.946, *p* < 0.001, η^2^ = 0.327. The comorbidity group had significantly longer total viewing time than the ADHD group (*p* = 0.007) and control group (*p* < 0.001). The difference between the latter two did not reach significance (*p* = 0.158). There was a significant main effect of the material type, *F*(4, 164) = 19.338, *p*_corrected_ < 0.001, η^2^ = 0.320. Participants’ total viewing time of target characters was longer than that of distractors (*p*s < 0.001). The total viewing time of orthographically similar characters was longer than that of homophonic, unrelated, and red characters (*p*s < 0.001), but the latter three conditions had no significant differences (*p*s > 0.05). The interaction between group and type was significant, *F*(8, 164) = 4.603, *p*_corrected_ = 0.011, η^2^ = 0.183. As shown in Figure 2, the simple effect test indicated that the total viewing time of the comorbidity group and ADHD group was longer than the control group in orthographically similar and homophonic conditions (*p*s < 0.05), whereas the two former have no significant difference (*p*s > 0.05). There was no group effect on unrelated characters (*p* > 0.05). Notably, the comorbidity group has longer total viewing time than the pure ADHD group (*p* = 0.002) and the control group (*p* < 0.001) in red-color condition, whereas the difference between the latter two groups did not reach significance (*p* > 0.05). In addition, the comorbidity group has longer total viewing time than the ADHD group (*p* = 0.014) and control group (*p* = 0.002) for the target character.

### Number of Fixations

The main effect of the group factor was significant, *F*(2, 41) = 3.442, *p* = 0.042, η^2^ = 0.144. Pairwise comparisons showed that comorbidity group was inferior than the ADHD (*p* = 0.031) and control groups (*p* = 0.027). There was also a significant main effect of the material types, *F*(4, 164) = 16.327, *p*_corrected_ < 0.001, η^2^ = 0.285. Participants’ number of fixations on target characters was significantly larger than that on the distractors (*p*s < 0.001), whereas the red character condition received the least number of fixations than other conditions (*p*s < 0.001). The interaction between group and type was significant, *F*(8, 164) = 4.162, *p*_corrected_ = 0.016, η^2^ = 0.169, but the simple effect test did not show any significant group effect for different types of distractors.

## Discussion

The present study aims to investigate whether the comorbidity of ADHD and DD has the same or different cognitive overactivity as pure ADHD has. As we intended to examine both attention/inhibition and language aspects simultaneously, the task of visual search for Chinese characters was conducted. Although visual search tasks have long been used to inform on the nature of selective attention in adults (Wolfe, 1998), their potential has not been fully realized in the field of ADHD research (Mullane and Klein, 2008).

Although the comorbidity group and the pure ADHD group did not differ from each other in terms of accuracy, the comorbidity group had a longer reaction time than the pure ADHD group, suggesting that reaction time may be more sensitive to distinguish these two groups. This is consistent with previous results: DD and ADHD both have cognitive deficits in processing speed (Willcutt et al., 2010). Although the most frequently assessed dependent variables for visual search are reaction time and accuracy rates (Wolfe, 1998), eye-movement recording and its measurements during visual search and reading may provide insight into the pathogenesis of ADHD and DD (Fabio et al., 2019; Mohammadhasani et al., 2019). For instance, although the first fixation duration reflects early processing and attention attraction on the stimuli, gaze duration and total viewing time indicate relatively late stage for information integration and attention maintenance (e.g., Inhoff, 1984).

For the early processing stage, eye-movement data show that the two ADHD clinical groups have similar patterns in the first fixation duration, and those groups are significantly slower than typically developing children in all types including unrelated characters. These findings are consistent with previous literature that supports the shared cognitive deficit model (Willcutt et al., 2005). As the first fixation duration is a relatively early (fast) measurement, which is sensitive to bottom-up visual features (Rayner, 1998), the finding of first fixation duration also suggests that the two ADHD clinical groups have shared impairments in automatic processes (Fabio, 2017; Martino et al., 2017; Caprì et al., 2020).

For the relatively late processing stage, the main group effect indicated that the comorbid group produced longer gaze duration and total viewing time on distracting characters relative to the pure ADHD group. The comorbid group also showed a higher number of fixations within characters and more interregional saccade number among characters, which are related to information integration. These results provide evidence for the distinguished cognitive deficit model, which proposes that the comorbidity pattern has a distinctive syndrome (Neale and Kendler, 1995). Specifically, children with comorbidity demonstrated a unique pattern of gaze duration and total viewing time in the red character condition deficit. This means that they had worse capacity to control their attention to accomplish the current task, making them more likely to be attracted by novelty (e.g., a distinct color). It has also been previously reported that individuals with comorbidity (ADHD + DD) perform poorer in color naming (e.g., Stroop task) than do individuals with pure ADHD (Rucklidge and Tannock, 2002). These findings indicate that the comorbidity (ADHD + DD) has a unique deficit in controlled processes and inhibition responses when compared with the pure ADHD and the control, which is also in line with the distinguished cognitive deficit model.

Relevantly, Mohammadhasani et al. (2019) compared the eye-movement measures between ADHD and typically developing children in a word memory test, in which participants were requested to view all the words (16 words arranged in a 4 × 4 matrix) in each trial and repeat the word list after each presentation. They did not find a reliable group effect on fixation length, but they observed atypical visual scanning for ADHD individuals. The present study also used linguistic materials (five characters in each presentation), but the participants did not necessarily view all the stimuli to find the target. We mainly focused on the results of fixation duration while the visual scanning was not analyzed owing to sparse fixations in each trial. The discrepancy in findings between the present study and the study of Mohammadhasani et al. (2019) may be attributed to different types of tasks or modes of attention shifting. Nevertheless, the current study and previous studies (e.g., Gould et al., 2001; Deans et al., 2010; Türkan et al., 2016; Mohammadhasani et al., 2019) have converged to show that individuals with ADHD have poor oculomotor control. Moreover, the present study has found that the deficit of ADHD can arise in the early processing stage and that the deficit of comorbidity (ADHD + DD) has a unique deficit in the late processing stage.

The comorbidity and pure ADHD group produced longer total viewing time in orthographically similar and homophone conditions relative to the control group, but there was no significant group difference in the unrelated condition. This result indicates that both of two clinical groups had deficits in language processing, which appeared in a relatively late stage. The deficits in switching or mapping between orthography and phonology may result in longer total viewing time on orthographically similar and homophonic characters.

The patterns of fixation duration and number of fixation both show that it is very difficult to reject the orthographically similar characters relative to homophones, unrelated, and red characters. This result indicates that in the visual search of characters, the identification of target characters is susceptible to orthography. In contrast, the red color was task irrelevant, which attracted the least number of fixations. Despite that, comorbid individuals spent a longer time on the red characters in the late processing stage, which reveals their deficit in inhibition.

Note that the present study has some limitations. Owing to the particularity of ADHD and comorbidity, it is relatively difficult to recruit participants, especially girls, who are less likely to be diagnosed and treated for ADHD symptoms than boys (Biederman et al., 2002; García, 2019; Slobodin and Davidovitch, 2019). Our study may lack the power to generalize a robust conclusion as a result of small sample size and gender bias. As the present study mainly focused on ADHD with or without DD, we did not include children with pure DD. However, future studies could include pure DD as an independent group to clarify the association and difference among ADHD, DD, and their comorbidity. As there is currently no standardized Chinese version of K-SADS-PL5, which serves as the complementary diagnostic tool for DSM-5, we used the DSM-IV (K-SADS-PL) for the diagnoses in this study (see section “Materials and Methods” for details). Although the changes in the diagnostic system (DSM-IV vs. DSM-V) would have limited effects on the diagnosis application for children with ADHD (van de Glind et al., 2014), using the DSM-5 as diagnostic criteria should be promoted in future studies.

Taken together, this study found that, in an early processing stage, both clinical groups of children showed a longer fixation duration in distractors than did the control group. The shared deficits in ADHD clinical groups might be explained by the deficits in automatic processes (Fabio, 2017; Martino et al., 2017; Caprì et al., 2020). However, the result of the late processing stage showed that children with comorbidity have a unique deficit in attention and inhibition. Therefore, the present study provides evidence for the shared cognitive deficit model in the early processing stage and for the distinguished cognitive deficit model in the late processing stage. We agree that a single model was not enough to explain the causes of comorbid DD and ADHD (Pennington, 2006; Willcutt et al., 2010), and multiple models may apply to different processing stages (Langer et al., 2019). The findings of the current study are important for the contribution to investigate visual search and lexical processing in clinical groups like comorbidity of ADHD and other disabilities, showing eye-movement measures may be helpful in the diagnostic procedure of comorbidity in the future. It is suggested to further explore the deficit mechanism of comorbidity children in future studies.

## Data Availability Statement

The datasets generated and analyzed during the current study are available from the corresponding author on reasonable request.

## Ethics Statement

The studies involving human participants were reviewed and approved by Ethics Committee of the Peking University Sixth Hospital. Written informed consent to participate in this study was provided by the participants’ legal guardian/next of kin.

## Author Contributions

WZ and YW conceptualized and designed the study and critically reviewed the manuscript for important intellectual content. YW diagnosed the ADHD patients. JW, MS, and YW coordinated and supervised the data collection. XC and YC performed the data collection and data analyses. XC drafted the initial manuscript. XC, JW, HS, and ZW reviewed and revised the manuscript. All authors approved the final manuscript as submitted and agree to be accountable for all aspects of the work.

## Conflict of Interest

The authors declare that the research was conducted in the absence of any commercial or financial relationships that could be construed as a potential conflict of interest.
